# Global spatiotemporal and genetic footprint of the H5N1 avian influenza virus

**DOI:** 10.1186/1476-072X-13-14

**Published:** 2014-05-21

**Authors:** Ruiyun Li, Zhiben Jiang, Bing Xu

**Affiliations:** 1 State Key Laboratory of Remote Sensing Science, College of Global Change and Earth System Science, Beijing Normal University, Beijing 100875, China; 2 School of Environment, Tsinghua University, Beijing 100084, China; 3 Department of Geography, University of Utah, Salt Lake City, UT 84112, USA

**Keywords:** Spatiotemporal, Genetic, Avian influenza virus, H5N1, Transmission footprint, Global

## Abstract

**Background:**

Since 2005, the Qinghai-like lineage of the highly pathogenic avian influenza A virus H5N1 has rapidly spread westward to Europe, the Middle East and Africa, reaching a dominant level at a global scale in 2006.

**Methods:**

Based on a combination of genetic sequence data and H5N1 outbreak information from 2005 to 2011, we use an interdisciplinary approach to improve our understanding of the transmission pattern of this particular clade 2.2, and present cartography of global spatiotemporal transmission footprints with genetic characteristics.

**Results:**

Four major viral transmission routes were derived with three sources— Russia, Mongolia, and the Middle East (Kuwait and Saudi Arabia)—in the three consecutive years 2005, 2006 and 2007. With spatiotemporal transmission along each route, genetic distances to isolate A/goose/Guangdong/1996 are becoming significantly larger, leading to a more challenging situation in certain regions like Korea, India, France, Germany, Nigeria and Sudan. Europe and India have had at least two incursions along multiple routes, causing a mixed virus situation. In addition, spatiotemporal distribution along the routes showed that 2007/2008 was a temporal separation point for the infection of different host species; specifically, wild birds were the main host in 2005–2007/2008 and poultry was responsible for the genetic mutation in 2009–2011. “Global-to-local” and “high-to-low latitude” transmission footprints have been observed.

**Conclusions:**

Our results suggest that both wild birds and poultry play important roles in the transmission of the H5N1 virus clade, but with different spatial, temporal, and genetic dominance. These characteristics necessitate that special attention be paid to countries along the transmission routes.

## Background

The hemagglutinin (HA) segment of H5N1 highly pathogenic avian influenza (HPAI) virus has been reported to have 10 distinguished clades ranging from 0 to 9 [1]. Among these, the Qinghai-like lineage (clade 2.2) was the quickest to evolve, with a fourth-order sub-clade up to the present. Moreover, of the many genetic clades circulating in Asia, clade 2.2 is considered the only one that spread westward to the Middle East, Europe and Africa [2], reaching a dominant role with global range.

Much previous research has been completed and is of considerable significance to our study. For instance, some research works studying the virus transmission at nationwide and continent-wide spatial scope have been conducted based upon phylogenetic evolution theory, especially during 2005–2006 when the situation was severe [3,4]. Moreover, new interdisciplinary efforts using a combination of geospatial informatics and bioinformatics approaches have been made to improve understanding of global H5N1 transmission [5,6]. However, some limitations of these works remain to be solved. First, most research has focused on certain regions or small spatial scales. For example, some works explore the transmission pattern only within a single country, such as India [7,8], Egypt [9], Mongolia [10], the Russian Federation [11], France [12], Germany [13], Switzerland [14], Burkina Faso [3], Hungary [15], Bangladesh [16], and Kuwait [17]. Other studies have remained focused within a specific continent, such as Africa [18,19]. The study of cross-continent spread [2] is even less common, not to mention examination of disease diffusion at a global scale, which probes the transmission footprints among H5N1-infected countries globally. In addition, other works concerning clade 2.2 routes are relatively approximate, and appear to have simplified the transmission pattern and underestimated the complexity of this clade.

Given the significance of the Qinghai-like lineage and lack of knowledge about its probable means of transmission, there is a need to investigate its geographic spread mechanisms through investigating its underlying genetic forcing. In this research, we examine potential global transmission routes during 2005–2011, and the spatiotemporal distribution features of outbreaks along these routes. We also explore genetic mutation features relative to the isolate A/goose/Guangdong/1996 (Gs/Gd/96), to enhance our understanding of clade 2.2 from a novel, macroscopic view.

## Methods

### Outbreak data

Global time-location series of H5N1 outbreaks, containing 11750 records from January 2005 through September 2011, were extracted from official reports of the Food and Agriculture Organization of the United Nations (http://www.fao.org). The associated variables and parameters of H5N1 outbreaks are outbreak location, observation and reporting date, and some descriptive information about the corresponding host. Incomplete records, especially those without location information, were labeled with Google Earth, and the center of the available administrative region where the outbreak occurred was used instead.

Outbreak data were used mainly to identify transmission direction, especially when temporal information was unavailable for genetic sequences. These sequence records were mainly from Egypt, Nigeria and Germany. The data were also used to analyze the spatial and temporal host distribution pattern along transmission routes.

### Sequence data

GenBank of the National Center for Biotechnology Information (NCBI) (http://www.ncbi.nlm.nih.gov) has collected and published HA and neuraminidase (NA) sequence data of HPAI clades. From the database website, we obtained global HPAI H5N1 full-length sequence data from 2005 to 2011. This selected time period for the sequence data was in accordance with that of the H5N1 outbreak data. In addition to the basic sequence information, year and location of the isolates and host data were also collected. In practice, many genetic fragments were incomplete. Therefore, only those segments with lengths greater than 1600 base pairs were selected, for a total of 873 sequence data records of HA clade 2.2. Accession numbers of the nucleotide sequences were provided (Additional file 1). Among the 873 sequences of the HA genes, 83 are from humans, 580 are from poultry, and 210 are from wild birds. The number of sequences obtained and used for each country is summarized in the Additional file 2.

Using Molecular Evolutionary Genetics Analysis (MEGA) software version 4.0 [20], the collected sequence data were initially aligned and compared. They were then combined with the sequences in a small tree from the World Health Organization (WHO) (http://www.who.int/en). The phylogenetic tree was constructed by both the maximum-likelihood and neighbor-joining distance-based matrix algorithms to ensure the identity of tree topology. For most sequences, spatial and temporal information was available on a country and year basis, respectively. Exceptions were some isolates in geographically large countries such as China and Russia, which had province-level information. In this way, source articles of all sequences were referred to and checked, to implement their detailed descriptions in the sequence database. With this dataset of improved accuracy, it was easier to match sequences with geospatial data based on their spatial and temporal attributes; nevertheless, some temporal information was unavailable and required supplementation by outbreak data in the following analysis. In addition, sequence data were used to calculate genetic distances to the Gs/Gd/96 isolate.

### Construction of routes and paths

The terms route and path are used to illustrate the overall and detailed transmission pattern of virus, respectively. The former depicts the broad transmission mode and the latter gives further regional spread information.

**Figure 1:**
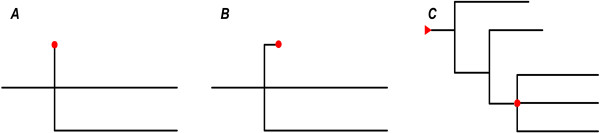
**Rule of root identification.** Red dot represents root of a group, and red triangle shows root of an entire tree. For a specific group, root identification depends on genetic distance of a virus strain to its father node. If there is a virus strain whose distance to the father node is **(A)** zero or **(B)** nearly zero, then this node is considered the root of the group because it has the closest distance to the father node relative to other viruses in the same group. **(C)** If no such node exists, the father node acts as the root, looping until the root of the entire tree is reached.

Generally, routes were constructed here in two steps; namely, 10 paths were identified based on topology of the phylogenetic tree, and these were then aggregated into four possible routes.The 10 paths, especially the transmission direction between countries, were identified using the following criteria. First, identification was done following the “time order” criterion; in other words, it is assumed that spread was from early-occurring viruses to those occurring later. In addition, we followed the “distance order” criterion. This is the assumption that the shorter the distance to the root, the higher probability a virus has to act as a source and infect others distant from the same root. Last, a rule was established to identify the root in the second criterion. Under certain circumstances, the chronological order of viruses within the same group (a set of isolates that share a parent node) may not be clear; thus, if the first criterion was not in effect then the second one, which requires definition of the root for a group, was followed. In this case, two situations and two corresponding solutions were found. Specifically, if there is a virus whose distance to the parent node is (or nearly) zero, then this node is considered the root of the group, since it has the closest distance to the parent node relative to other viruses in the same group (Figure 1A and B). If this is not the case or no such node exists, then the parent node is regarded as the root of the group, and thus the parent node of this parent node, looping until the root of the entire tree is reached (Figure 1C).

According to the geographic locations of the studied countries in each group, 10 paths were integrated into four possible routes, based on the presumption that there may be certain similarities among genetic sequences in paths within the same countries.

### Calculation and spatial distribution of genetic distance

Genetic distances to the Gs/Gd/96 isolate were calculated with the Kimura two-parameter model [20] in MEGA version 4.0, using a gamma distribution (shape parameter = 1) to model rate variation among sites. Results were stored in matrix form.

The average genetic distance to Gs/Gd/96 along the *i*_*th*_ route, *D*_*i*_, represented the mean genetic mutation among all regions on the *i*_*th*_ route. The average genetic distance to the Gs/Gd/96 isolate in the *j*_*th*_ region on the *i*_*th*_ route, *D*_*ij*_, indicated mean genetic mutation within the *j*_*th*_ region on the *i*_*th*_ route. If *D*_*ij*_ > *D*_*i*_, the *j*_*th*_ region was considered to have a positive role in genetic mutation on the *i*_*th*_ route, or be a “key” region, and its geographic location was marked for further analysis of the spatiotemporal distribution of genetic mutation along routes.

## Results

### Paths and routes

**Figure 2:**
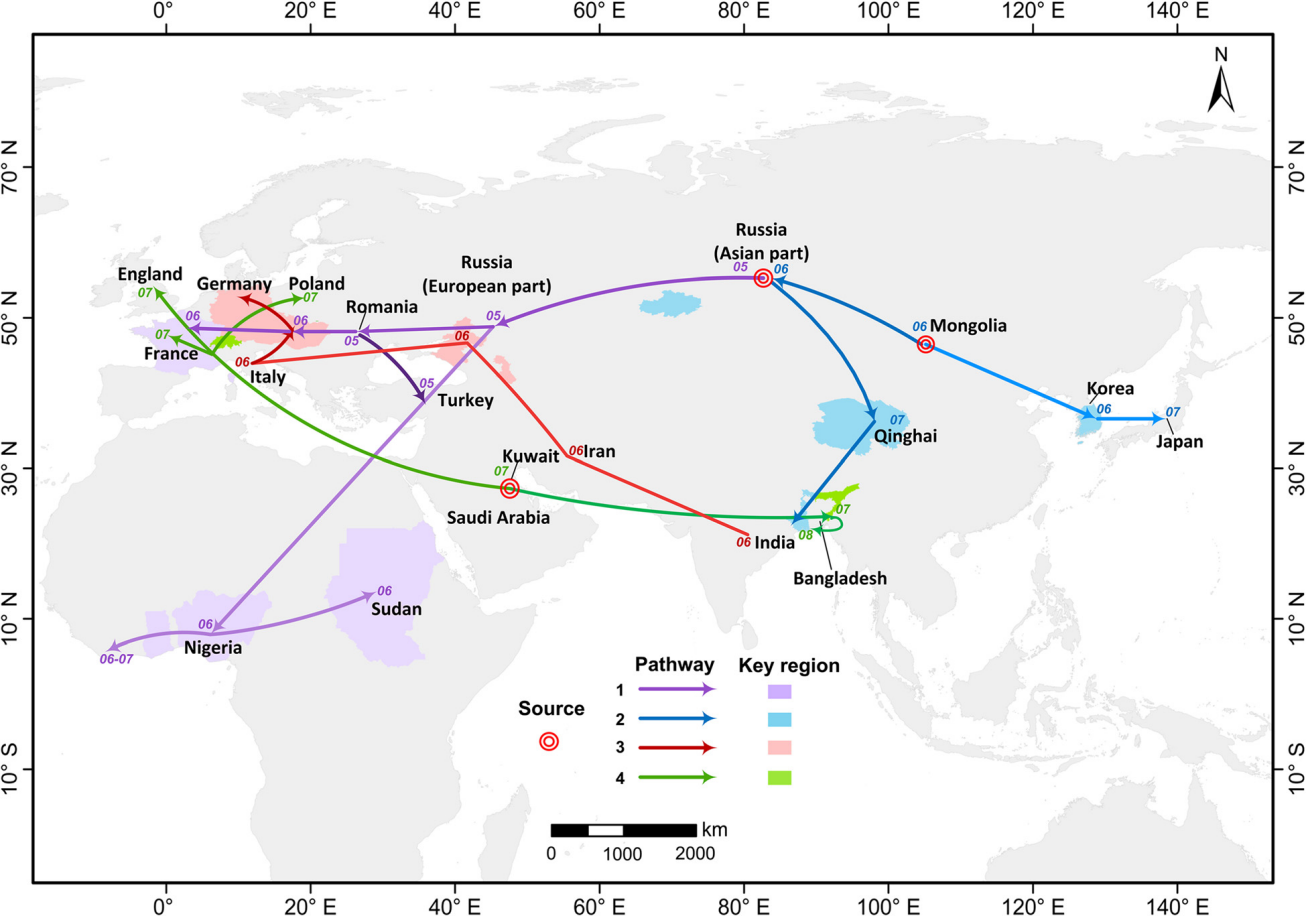
**Four major global transmission routes of clade 2.2H5N1 avian influenza.** Each color represents a single route with its corresponding key regions. Colored patches show regions that have larger genetic distances along the route. Three probable sources, namely, Russia, Mongolia and the Middle East (Kuwait and Saudi Arabia) in three consecutive years (2005, 2006 and 2007), are indicated by a red, ring-shaped symbol.

Details of the 10 paths and four possible routes are shown in Table 1, and the corresponding spatial attributes are depicted in Figure 2. Arrow shapes in Table 1 are used to represent the criteria obeyed in identifying the transmission direction between countries or regions. No clear directions were found among countries in the seventh path and the first half of the third route. Spatially, clusters along the same route have the same color system but varying colors, which illustrates not only the small difference (or certain similarities) among those within-route paths, but also large among-route distinctions.

**Table 1:** Detailed transmission paths

**Path**	**Directions**	**Route**
***a***	05.2 Russia (Asian) → 05.11-06 Russia (European) → 05 Romania → 06.2 Hungary & 05 Croatia	***1***
***b***	05 Russia (European) → 06 Nigeria → 06 Sudan & 06–07 West Africa	
***c***	05 Romania & 05 Turkey → 06.2 Austria & 06.2 Slovenia → 06 France	
***d***	05 Romania & 05 Turkey → 06 Nigeria	
***e***	06.5 Mongolia → 06 Korea → 07 Japan	***2***
***f***	06.5 Mongolia → 06.6 Russia → 07.4 Qinghai → 08 India	
***g***	India -- Iran -- Russia -- Italy	***3***
***h***	06.2 Italy → 06.2-3 Slovenia & Austria & 06.2 Hungary & 06.2 Slovakia → 06.4 Czech → 06 Germany	
***i***	07 Kuwait & 07 Saudi Arabia → 07-11Bangladesh → 08–10 India	***4***
***j***	07 Kuwait & 07 Saudi Arabia → 07 Germany → 07–08 other European countries	

Arrow indicates transmission direction between two countries. Dashed line indicates that the direction of transmission is uncertain. The infected date is provided afore the country name.

### Characteristics of spatiotemporal transmission

The first route is probably the most complex, and consists of four paths. This route began from the Asian part of Russia in 2005 and initially spread westward within that country, reaching its European part in late 2005. After this within-country spread, transmission split in two directions, westward and southwestward. The former trajectory reached France through the southern part of Austria, Slovenia, Croatia and Hungary in 2006. In the latter course, viruses from Romania and Turkey combined in 2005 and spread to Africa in 2006 or, more precisely, to Nigeria in February 2006. Transmission continued west and east, affecting western African nations and Sudan in the east. Thus, western Asia and eastern Europe may be sources for Africa.

The source of the second route is viruses isolated in Mongolia during May 2005, which then followed two distinct paths. One of these impacted Korea and Japan, and the other path spread westward to the Asian part of Russia, and then southward to Qinghai, China, and India over the two years, constructing links among Mongolian, Russian, Qinghai, and Indian viruses during May 2005 through 2008.

In the third course, the direction of transmission between countries in the first half is unclear, but shows potential relationships between viruses in southwest Asian and European countries. However, in the second half of this route, viruses originated in Italy in February 2006, affecting central and southern European countries such as Hungary, Slovakia, Slovenia and Austria almost simultaneously. Several months later, the Czech Republic and Germany were affected. Based on this transmission route, Italy may be an origin for those central and southern European countries.

The last footprint shows a distinct time and source feature relative to the aforementioned ones, i.e., many viruses in this pathway were isolated in 2007, with a source in southwest Asia (Kuwait and Saudi Arabia). This route has two sections; specifically, one spread east to Bangladesh and India and another transect crosses western Europe, affecting France, Austria, Germany, Poland, the Czech Republic, and the UK. Thus, southwest Asia may be a source for the viruses in Europe.

Based upon the description above, we found three main sources; namely, the Asian part of Russia, Mongolia, and southwest Asia (Kuwait and Saudi Arabia), which have clear spatial and temporal features. In particular, the first two were responsible for the 2005–2006 transmission and are in the northern part of Asia, whereas the third and fourth in southwest Asia were related to spread in 2007 and 2008. European countries and India had at least two virus incursions along multiple routes, producing a mixed situation.

### Spatial features of genetic mutation

The key regions of genetic mutation are illustrated in Figure 2. It is clear that nearly all are located at the end of each path, which means that with spatiotemporal transmission along the route, genetic distances relative to Gs/Gd/96 are becoming much greater. This indicates an ongoing evolution of viruses, causing a precarious situation in certain regions such as Korea, India, France, Germany, Nigeria, and Sudan.

### Spatiotemporal distribution pattern of hosts

**Figure 3:**
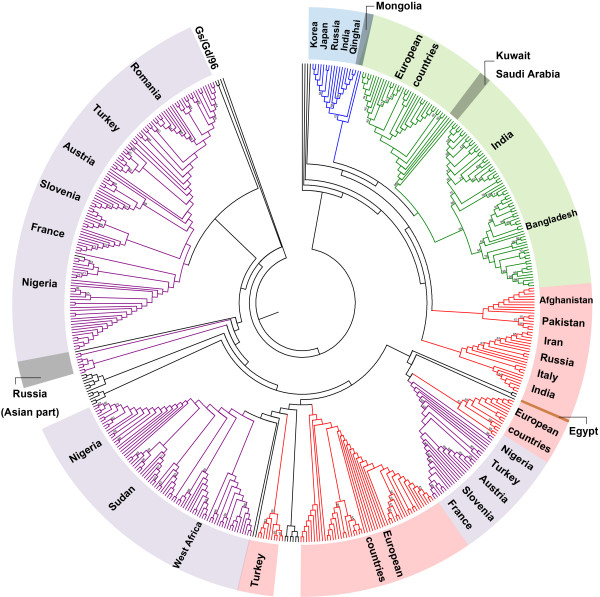
**Phylogenetic analysis of HA segment of clade 2.2 H5N1 influenza viruses.** Topology of phylogenetic relationship of studied 873 HA genes in four routes is shown by different color associated with its corresponding route. The phylogenetic tree is rooted by Gs/Gd/96 isolate with three possible sources shown in grey with country names annotated. Bootstrap values greater than 60 are marked at corresponding branches.

**Figure 4:**
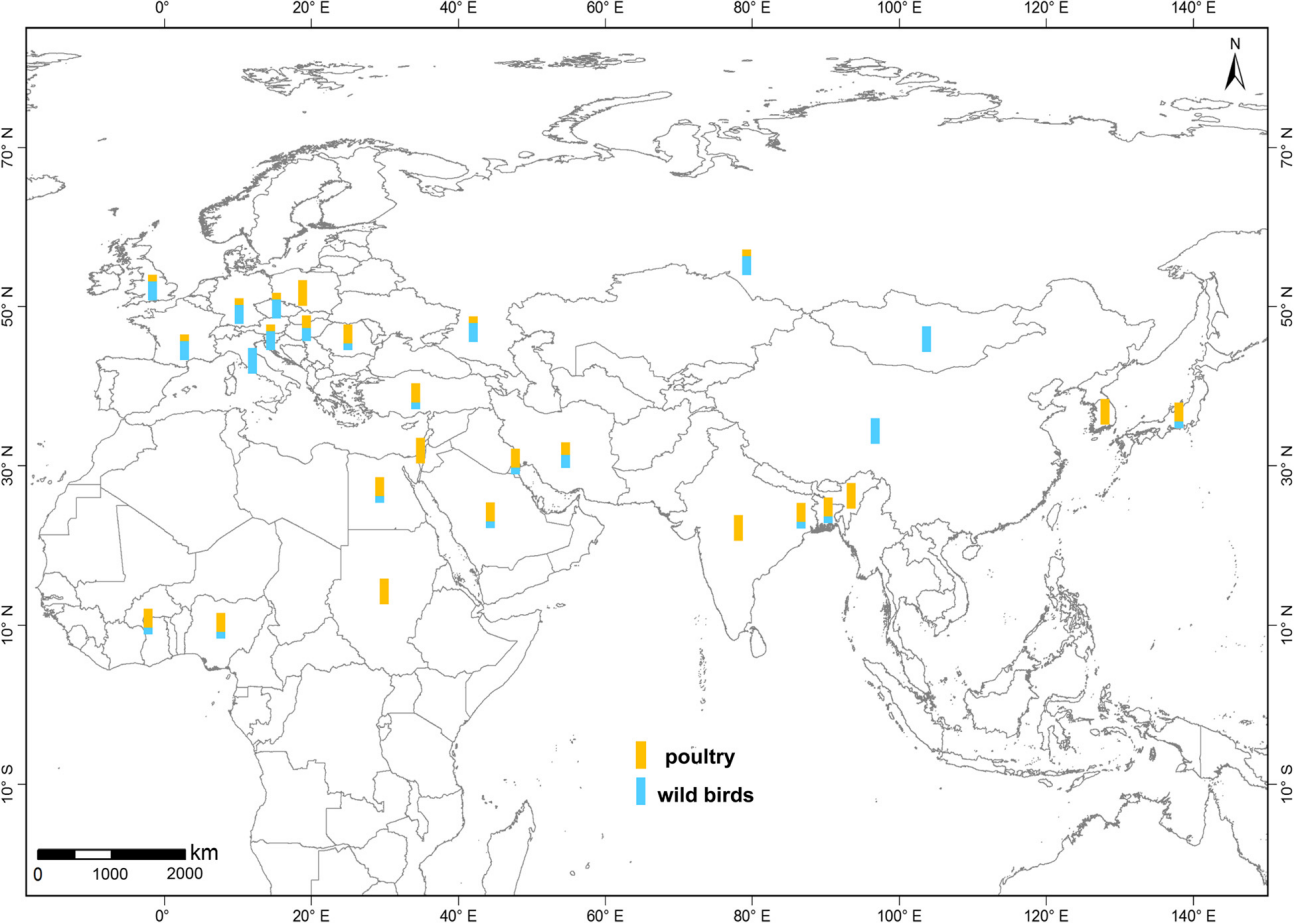
**Spatial distribution of infected wild birds and poultry along four transmission routes.** Isolates from wild birds and poultry are shown respectively in blue and yellow bars, with bar height representing proportion of isolates in the hosts of wild birds and poultry.

**Figure 5:**
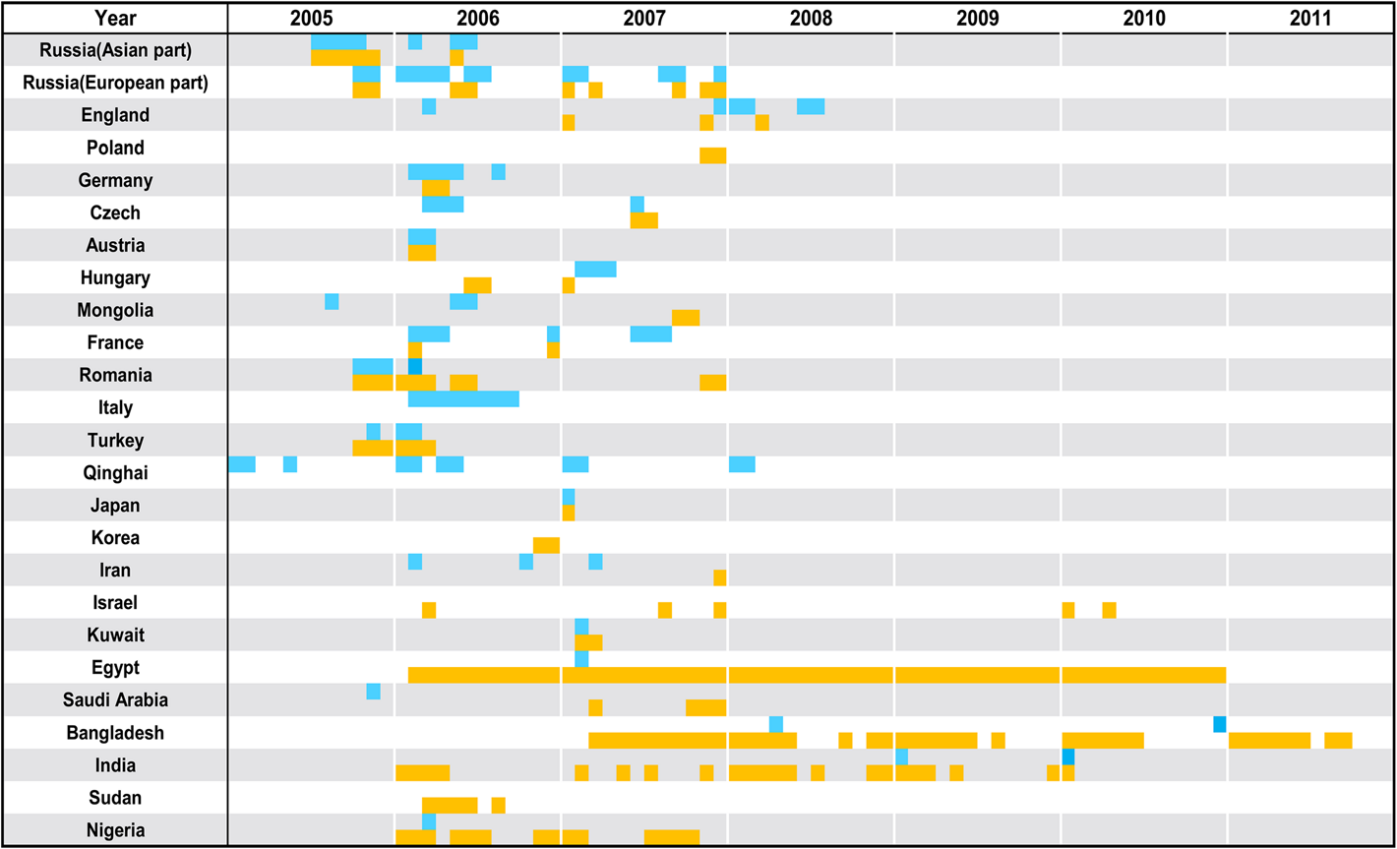
**Timeline of emergence of clade 2.2 H5N1 influenza virus.** A list of countries are ordered from higher latitude to lower latitude ones. Isolates from wild birds and poultry are labeled in blue and yellow squares, respectively. The location of the bar indicates the month of the outbreak occurred and its length reflects the corresponding duration (in months) of the outbreak.

The distribution of hosts in clade 2.2 transmission paths shows clear spatial and temporal patterns, with 2007/2008 as the important separation point (Figure 3 and 4). Spatially, viruses isolated from poultry are mainly in East and South Asia, the Middle East and Africa, with some sporadic occurrence in Central Europe such as in Poland and Romania. For viruses isolated from wild birds, the majority are in Europe and certain parts of Asia (Qinghai, China and Mongolia), with sparse incidence in Africa and some areas of Asia. Temporally, viruses that were present in poultry lasted nearly through the entire period of 2005–2011. In particular, India and Bangladesh were most affected during 2007–2011. Noncommercial poultry operations, such as backyard poultry facilities might be potential sources of virus exchange between commercial poultry and wild birds. This is particularly critical in wetland areas of southern and southeastern Asia where backyard poultry have close contact with commercial poultry and migratory birds, therefore increasing the risk of contracting disease [21]. In contrast, wild bird viruses were mainly present during 2005–2007, and especially in 2006, after which there were only very rare outbreaks in four countries: China, India, Bangladesh and the UK. In summary, wild birds were the major vector for the spread of viruses during 2005–2007, and caused global infection and virus transmission. On the other hand, poultry may have been responsible for the regional virus transmission of 2008–2011.In Figure 5, countries are arranged by latitudinal order, and there is clear geo-temporal separation or a high-to-low latitude transmission pattern. That is, viruses isolated from wild birds were clustered around 30°N and in northern regions before 2007; those isolated from poultry were largely further south, in Egypt, Nigeria, India, Bangladesh and other countries, especially after 2007.

## Discussion

### Transmission directions and sources

We summarized four major transmission routes of the clade 2.2 virus with three sources; namely, Russia, Mongolia and the Middle East (Kuwait and Saudi Arabia). Based on the proposed criteria, we were able to establish clear transmission directions between countries on three routes, not just the similarities indicated by previous works such as Ducatez et al. [19].

The east-spreading section to Bangladesh and India in the last route may indicate that viruses in India during 2008 may be more precisely from Kuwait rather than Bangladesh, as previously assumed [22]. In addition, European countries had at least two virus incursions with distinct spatiotemporal characteristics. Previous findings that suggest differences between viruses isolates before and after 2007 [13,22-24], even in the same country, may confirm the present result that European viruses may have had two separate sources and routes in different years.

### Role of Indian and Italian viruses

In the foregoing analysis, India has two different sources and Italy is recognized as one source for Europe. Nevertheless, given the uncertainty of transmission direction in the first half of the third route, there may be underlying relationships between southwest Asian and European countries. In this case, India may have been the source or pool of viruses in 2006, but strong evidence of this is unavailable. It is likely that one or more countries on the first half of this route may have acted as the source in a more exact sense. Thus, whether Italy should be treated as a source requires more consideration.

### Egyptian isolates

It was found that Egypt had only a single virus introduction and was declared endemic in 2008. The origin or source of these Egyptian isolates has not been identified, although some research has indicated that they are of Eurasian origin [25]. The present focus is on transmission routes, therefore the sources of these isolates are important. Thus, the Egyptian isolates are not included in any path, but the lack of these isolates in our four routes does not mean that they are not crucial to maintenance and mutation of the virus.

### Intersection and similarity of routes

As seen in Figure 3, isolates from along the four transmission routes are not completely mutually independent. There is some intersection, especially among viruses isolated in Nigeria, Turkey, Austria, Slovenia, and France in the first path, and those in European countries in the second half of the third route. Further, both routes eventually entered the European countries at nearly the same time. This spatial and temporal similarity between the two may be responsible for the intersection between isolates on the two paths.

There are some similarities between the transmission mode of the first clade 2.2 route and the global spread process of H5N1 outbreaks [18]. That is, routes of clade 2.2 that spread westward to Europe and southward to Africa may be identical to outbreak trajectories in these two directions. This may be because that the west- and south-spreading viruses from eastern Europe during 2005–2008 all belong to the 2.2 clade, or that this clade is the only one that appears during this period and along those transmission directions.

### Host issues

As mentioned above, the first route covers the largest geographic area relative to the other three. This large spatial extent was generally facilitated by high infectivity and a long infectious period [26]. In our study, chickens, ducks and geese were the primary victims and they had a wider spatial distribution than other host species, which indicates greater infectivity. Moreover, migration connects many bird populations in time and space along major flyways, either at common breeding areas during migration, or at stopover and wintering sites. This provides ideal places for many species to aggregate at high local density, causing cross-species infection. Also, virus-infected birds may transmit pathogens to other populations that may subsequently move to new areas [27]. This forms a “transmission relay” with a long and continuous infectious period, which may reflect the importance of migratory birds in the perpetuation and spread of H5N1 viruses. The combination of experimental exposure data and telemetry information has been used in previous works to describe the intercontinental virus dispersion by a series of successively infected migratory birds [28]. In addition, experimental studies have shown that several bird species survive infection and shed the H5N1 virus without apparent disease signs. Even worse, HPAI may retain high pathogenicity in chickens, in contrast to the lower pathogenicity found in infected ducks [29]. This asymptomatic spread in birds and pathogenicity differences in hosts may partly explain the circulation of viruses and high death rate among chickens in Nigeria.The emergence timeline of clade 2.2 (Figure 5) shows only the emergence time of the clade in each country during 2005–2011, and is distinguished by poultry and wild birds. However, the number of outbreaks in each month is not shown. Therefore, the difference in seasonality of outbreak intensity between countries or hosts (poultry and wild birds) is not reflected.

Analysis of spatiotemporal distribution of various hosts along routes is done at the wild bird and poultry level. Moreover, roles are addressed in light of amounts and relative ratios of the two hosts in various spaces. These analyses have limitations. First, humans are not taken into consideration. H5N1 clade 2.2 human cases are common, especially in Egypt, and sporadic viruses have been isolated in Turkey, Azerbaijan, Iraq and other places. Despite barriers to transmission from donors to recipients, factors like human population growth, travel, and the legal and illegal poultry trade gradually weaken these geographic and behavioral barriers [26]. This increases direct avian-to-human transmission with no recombination or apparent mutation [30], generating many human H5N1 cases such as in the severe situation of Hong Kong in 1997. However, this transmission does not mean that human-to-human transmission is also common. This transmission is likely limited to relatives, with no evident geographic clusters [31]. The characteristics of not only donors and recipients, but also emerging viruses, should be considered regarding human transmission patterns [26]. There has been no more detailed classification of host species, which may neglect the expanding host range of H5N1 viruses and newly infected host species. Pigs, for instance, are considered candidates for generating reassortant strains and a subsequent pandemic cause, because they have both avian and human influenza sialic acid residue receptors in their respiratory tracts [32]. Given data imperfections at the host species level, we cannot address the issue of whether the host range has expanded and pig-infecting viruses of clade 2.2 have occurred. Finally, as mentioned by Andrew and Glass [33], new host populations in which the virus is endemic represent new risks of pandemic influenza in human beings, since the avian strain is present year-round and may coincide with any human influenza season. Based upon the above analysis, Egypt declared the virus endemic in 2008, after having had a large number of H5N1 clade 2.2 human cases during 2005–2011 (a new host population was isolated in 2006 for the first time). Therefore, we infer that there may be a new risk of pandemic influenza in humans in Egypt.

### Data limitations

Owning to the under-reporting and general bias in the database considered, the four major global transmission routes presented here are possible scenarios based on evidence provided by our analysis. Thus, a well-established and shared database of H5N1 outbreaks and sequences is vital for further study and surveillance.

## Conclusions

We located four major transmission routes of the clade 2.2 H5N1 virus with three sources; namely, Russia, Mongolia, and the Middle East (Kuwait and Saudi Arabia) in three consecutive years of 2005, 2006, and 2007. With spatiotemporal transmission footprints along each route, genetic distances to A/goose/Guangdong/1996 virus are becoming significantly larger, leading to a more challenging situation in certain regions such as Korea, India, France, Germany, Nigeria, and Sudan. Europe and India have had at least two incursions along multiple routes, causing a mixed virus situation. In addition, spatiotemporal distribution along the routes showed that 2007/2008 was a temporal separation point for the infection of different host species; specifically, wild birds were the main host from 2005 to 2007/2008 and poultry was responsible for the genetic mutation in 2009–2011. “Global-to-local” and “high-to-low latitude” transmission footprints have been observed. Thus, both wild birds and poultry play important roles in transmission of the virus, but with different spatial, temporal, and genetic dominance. Special attention should be paid to countries along these transmission routes.

## Competing interests

The authors declare that they have no competing interests.

## Authors’ contributions

BX conceived the original idea. RL, ZJ implemented experiments. RL, ZJ, BX contributed to analyzing the experiments and writing the manuscript. All authors have read and approved the final version of this manuscript.

## Supplementary Material

Additional file 1 **Accession numbers of nucleotide sequences in the research.** 873 nucleotide sequences of HA clade 2.2 used in this work are downloaded from GenBank. Click here for file

Additional file 2 **The number of sequences obtained and used for each country.** The 873 sequence records of HA genes of clade 2.2 are distributed in 43 countries. Click here for file
